# “I just feel comfortable out here, there’s something about the place”: staff and client perceptions of a remote Australian Aboriginal drug and alcohol rehabilitation service

**DOI:** 10.1186/s13011-017-0135-0

**Published:** 2017-12-06

**Authors:** Alice Munro, Julaine Allan, Anthony Shakeshaft, Courtney Breen

**Affiliations:** 1 National Drug and Alcohol Research Centre, University of New South Wales, Sydney, NSW Australia; 2 Lyndon, Orange, NSW Australia

## Abstract

**Background:**

The need for effective, culturally safe residential rehabilitation services for Aboriginal people is widely acknowledged, however the combination of treatment components that is optimally effective, is not well defined. Most existing Aboriginal residential rehabilitation research has focused on describing client characteristics, and largely ignored the impact of treatment and service factors, such as the nature and quality of therapeutic components and relationships with staff.

**Methods:**

This qualitative study was undertaken as part of a three-year mixed methods community-based participatory research (CBPR) project that aimed to empirically describe a remote Aboriginal drug and alcohol rehabilitation service. Researchers utilised purposive sampling to conduct 21 in-depth, semi-structured interviews. The interviews used a ‘research yarning’ approach, a form of culturally appropriate conversation that is relaxed and narrative-based. The interview transcripts were thematically coded using iterative categorization. The emerging themes were then analysed from an Interpretative Phenomenological Analysis, focusing on how participants’ lived experiences before and during their admission to the service shaped their perceptions of the program.

**Results:**

A total of 12 clients (mean age 35 years, SD 9.07, 91% Aboriginal) and 9 staff (2 female, 7 male, mean age 48 years, SD 8.54, 67% Aboriginal) were interviewed. Five themes about specific program components were identified in the interview data: healing through culture and country; emotional safety and relationships; strengthening life skills; improved wellbeing; and perceived areas for improvement. This research found that Aboriginal drug and alcohol residential rehabilitation is not just about length of time in treatment, but also about the culture, activities and relationships that are part of the treatment process.

**Conclusion:**

This study highlights that cultural elements were highly valued by both clients and staff of a remote Aboriginal residential rehabilitation service, with the country or location being fundamental to the daily practice of, and access to, culture. Developing reliable and valid assessments of the program components of culture and treatment alliance would be valuable, given this study has reinforced their perceived importance in achieving positive treatment outcomes. Further, strengthening the aftercare program, as part of an integrated model of care, would likely provide greater support to clients after discharge.

## Background

The harmful effects of substance misuse on Australian Aboriginal and Torres Strait Islander individuals, families and communities (hereafter Aboriginal Australians as the term recommended by the Aboriginal Health and Medical Research Council for New South Wales; [2]) arises from a complex milieu of factors including the intergenerational impacts of colonisation [66], and subsequent high rates of incarceration [53], suicide and self-harm [26], and poverty [4, 37]. Despite Aboriginal Australians comprising only approximately 3% of the Australian population [3], drug and alcohol-related morbidity and mortality are disproportionately higher among this population [6, 7]. In order to significantly reduce the rates of harm, a range of effective prevention and treatment programs are required, including residential rehabilitation services.

Although the need for effective, culturally safe residential rehabilitation services for Aboriginal people is widely acknowledged, the specific combination of treatment components that is optimally effective is not well defined [33], under review; [20, 21, 31]. Research with mainstream drug and alcohol residential rehabilitation services, conversely, has consistently identified that length of stay is significantly associated with positive post-treatment outcomes [16, 17, 19, 24, 40, 45, 64]. Alternatively, the stage of treatment achieved by the client may provide another indicator of individual progress, but content of the stages may vary between different services. Toumbourou et al. [62] suggest that progress in treatment rather than length of time in treatment may be more predictive of improvements in a client’s level of function afterwards. However, ways to measure progress have not been described and failure to complete treatment is common [17]. A systematic review of studies published between 1992 and 2013 aimed at identifying risk factors for non-completion in mainstream residential rehabilitation found that 91% of studies exclusively focused on patient characteristics, such as age, sex, ethnicity, primary substance, marital status and co-occurring diagnosis, rather than people’s experience of specific treatment components, such as the perception of therapeutic activities and relationships with staff [17]. This review surmised that the key reason for the focus on client characteristics is the dominant medicalised understanding of substance misuse. From a medical perspective, failure to complete treatment is often viewed as the result of an underlying pathology, and therefore future research should focus on treatment-related components associated with failure to complete treatment in residential rehabilitation settings, in addition to patient characteristics [17, 30]. In settings other than residential rehabilitation, treatment components associated with improved retention include: the quality of the client-staff relationship, or therapeutic alliance [17, 41, 50]; ward atmosphere [19, 43]; a balanced treatment program [41]; and consistent daily routine [59].

Aboriginal-specific drug and alcohol residential rehabilitation services provide treatment for Aboriginal people with substance misuse problems. Since being established over five decades ago, Aboriginal residential rehabilitation services are reported to be the preferred option for Aboriginal people and have historically incorporated Therapeutic Community and 12-Step approaches, or a combination of both [15]. Aboriginal residential rehabilitation programs have an important role in responding to substance use disorders because they provide culturally specific services over an extended period of time, including providing a drug and alcohol-free environment, safe accommodation, time away from chaotic environments, access to counselling, and meeting clients’ nutritional needs [13, 15, 44]. Further, the National Indigenous Drug and Alcohol Committee suggests that residential treatment may be the best, or only practical, option for people who have a range of complex co-occurring needs [48].

An important component of Aboriginal residential rehabilitation services is ‘culture as treatment’ for healing from substance misuse [14, 15, 20, 61]. Indigenous peoples in Australia and internationally, perceive the aetiology of substance misuse and ill health in the erosion of their cultural integrity [14, 15, 22, 55, 20, 23, 63]. Re-connection with culture is, therefore, viewed as essential to recovery and ongoing wellbeing [22, 28, 46, 61, 63].

This study aims to identify staff and client perceptions of the key strengths and areas for improvement of specific treatment components delivered by a remote Australian Aboriginal drug and alcohol residential rehabilitation service.

## Methods

### Ethics

Ethical approval was provided by the Aboriginal Health and Medical Research Council and the University of New South Wales Human Research Ethics Committees (HC14142, 1023/14). All participants were provided with an information sheet explaining the purpose of this study, had the opportunity to ask questions, and signed a consent form to participate in the interviews. The study adhered to the principles outlined in National Health and Medical Research guidelines [47] and the Australian Institute of Aboriginal and Torres Strait Islander Studies Guidelines for Ethical Research in Indigenous Studies [5].

### Study context

Orana Haven Drug and Alcohol Residential Rehabilitation Service (OH) is an Aboriginal residential rehabilitation service located in Western NSW, 700 km northwest of Sydney. The service began operating as an Aboriginal Community Controlled Health Organisation (ACCHO) in 1983. It offers a 3-month voluntary rehabilitation program for predominantly Aboriginal males. OH’s current service provision is a combination of a Therapeutic Community and 12-Step treatment approach. According to OH’s 2015–2018 Strategic Intent, the service’s vision is to provide a culturally safe drug and alcohol healing centre that maximises the strengths of Aboriginal people and communities.

### Study design

This study was undertaken as part of a three-year (2014–2017) mixed methods community-based participatory research (CBPR) project that aimed to empirically describe a remote Aboriginal drug and alcohol rehabilitation service. CBPR typically involves cycles of collaborative action, often in sequential steps that engage community or service provider participants as co-researchers, educating and empowering them to effect positive changes in their environment [35, 36, 68]. The theoretical perspective of Interpretative Phenomenological Analysis (IPA) informed this qualitative study. IPA is based on the assumption that participant perceptions present evidence as their “lived experience” [57], with lived experience defined as the way a person experiences their world [9]. It has been previously argued that substance misuse is a lived experience rather than a behaviour and, therefore, it is legitimately studied within the context of the whole person [34]. Given this, IPA provides an appropriate methodology to interpret the meanings clients and staff ascribe to their lived experiences of OH [12].

### Participants and data collection

Researchers utilised purposive sampling [8] to conduct 21 in-depth, semi-structured interviews. The sample consisted of 12 clients (denoted in quotes by ‘C’) and 9 OH staff (denoted by ‘S’) attending or employed at OH from October 2015 to January 2016. Interviews were conducted across two different time points (less than 3 months apart) to ensure data collected reflected a range of client and staff experiences. The semi-structured interviews used a ‘research yarning’ approach, a form of culturally appropriate conversation that is relaxed and narrative-based [10]. Participants were initially asked about their life history (e.g. age, family, education, and occupation) prior to being admitted to, or employed at, OH. This enabled rich accounts of their experiences in a non-threatening way. Clients were additionally asked about the process of their referral to and length of time at OH. Participants were then asked a series of open questions about their experiences of OH (Table 1). Interviews were conducted in person at OH by a non-Aboriginal female researcher, took 30 minutes to 2 hours, were digitally recorded and transcribed by an independent third party into word documents. To ensure accuracy, the interviewer selected 10% of the interviews to re-transcribe and compare with the transcribed interviews.

**Table 1:** Summary of qualitative interview schedule for Orana Haven staff and clients

Questions and the order in which they were asked
Client interview schedule
1. What is your personal background/story?
2. Describe your health and life before you came to OH
3. What were your first impressions of OH?
4. What are your thoughts about your experience of OH once you had settled after a few days?
5. What has your mental and physical health been like since you have been at OH?
6. What activities have you been doing since you arrived at OH?
7. What are the cultural aspects of OH?
8. What are the next steps/plans for you after your discharge from OH?
9. What could OH do better?
10. Other comments to add?
Staff interview schedule
1. What is your personal background/story?
2. What is your role on a typical day at OH?
3. What happens when a client first arrives at OH – what is your role in the intake process?
4. What is OH’s approach for working with clients?
5. What are the cultural aspects of OH?
6. What happens when a client is getting ready to leave OH – what’s your role in the discharge process?
7. What could OH do better?
8. Other comments to add?

### Data analysis

The interview transcripts were deductively coded guided by the interview schedule (Table 1) and best practice recommendations for Aboriginal drug and alcohol residential rehabilitation services outlined by Brady [14] and also inductively coded into emerging categories by the first author (AM) using NVivo data management software (QSR International Pty Ltd). Then, iterative categorization [49], a systematic technique for managing analysis that is compatible with, and can support, analytical approaches such as thematic analysis, was used to deepen the analysis process by coding the categorised data line by line to identify emerging themes. The second author (JA) reviewed the coding for consistency. The emerging themes were then analysed from an IPA perspective, focusing on how interview participants ascribed meaning to their experiences and relationships within their environments. In this case, IPA prioritised the participants’ lived experiences before and during their admission to OH and examined how specific treatment components shaped their perceptions of the program [57].

## Results

### Sample

#### Client characteristics

The mean age of clients was 35 years (SD 9.07), most of whom identified as Aboriginal (91%, *n* = 11). On average, clients had been at OH for 48 days (range 4–120 days, SD 34.09). Over half were referred from the criminal justice system and reported prior substance rehabilitation experience, with a quarter indicating a previous admission to OH. All clients reported a history of incarceration. Most clients had children and two said they had a current partner. No clients reported completing high school education, although half had completed a trade qualification. All clients described chronic polysubstance substance misuse (predominantly methamphetamine, alcohol, cannabis and benzodiazepines), current mental health problems (e.g. psychosis, self harm, suicidality and depression) and extensive trauma in childhood or adolescence, ranging from one-off events to prolonged exposure. The types of trauma disclosed included: sexual abuse; being removed from their families and placed in foster care; family violence; parental substance abuse; and/or the death of a parent, sibling or child (Table 2).

#### Staff characteristics

Two female and seven male staff were interviewed. The mean age of the interviewed staff was 48 years old (SD 8.54) and the majority identified as Aboriginal (67%, *n* = 6). The average length of time that staff had been employed at OH was 2.6 years (SD1.43). Most were employed in full-time roles: two in management; two drug and alcohol counsellors; four residential care worker/other support roles (e.g. cook, maintenance, administration); and one in a dual transport and cultural advisor role (e.g. teaching woodwork, going on bush trips, taking clients to cultural sites). Two thirds of staff reported a history of substance misuse, of which over half stated they had had a prior drug and alcohol residential rehabilitation admission. Most reported a history of trauma in childhood or adolescence, including parental substance abuse or neglect, family violence and sexual abuse (Table 2).

**Table 2:** Summary of key client and staff characteristics

Characteristics	Clients	Staff
	N (%)	
Total participants	12	9
Age (mean, years)	35 (range 21–51, SD 9.07)	48 (range 36–61, SD 8.54)
Aboriginality	11 (91)	6 (67)
Gender		
Male	12 (100)	7 (88)
Female	n/a	2 (22)
Referral type		
Criminal justice	7 (59)	n/a
Self-referred	3 (25)	n/a
Other services	2 (17)	n/a
Length of time in treatment (mean, days)	48 (range 4–120, SD 34.09)	n/a
1–30 days	5 (42)	n/a
31–59 days	2 (17)	n/a
60–90 days	4 (33)	n/a
91 > days	1 (8)	n/a
Length of time employed at OH (mean, yrs)	n/a	2.6 (range 1–5, SD 1.43)
Prior incarceration	12 (100)	5 (56)
Prior substance misuse	12 (100)	6 (67)
Prior residential rehabilitation admission	7 (58)	5 (56)
Self-reported mental health issues	12 (100)	n/a
Self-reported history of trauma	12 (100)	6 (67)

### Emergent themes

Five themes about specific OH program components were consistently identified in the interview data: 1. Healing through culture and country; 2. Emotional safety and relationships; 3. Strengthening life skills; 4. Improved wellbeing; and 5. Perceived treatment gaps. The themes are detailed below.

### Healing through connection to culture and country

The presence and value of Aboriginal culture embedded throughout the OH program was described by staff and clients as a primary strength of the program. It was identified that providing ready access to culture by being on country directly impacted on people’s identity and spirituality. The term “country” is often used by Aboriginal and Torres Strait Islander people to describe the complex and interrelated connections to family origins and particular pieces of land in Australia and the Torres Strait [54].

#### Providing access to culture and country

Although there are many expressions of connections to culture among different Aboriginal communities, one common element is the concept of being ‘on country’. The specific nature of being on country varies depending on clients’ traditional lands, but the core construct of being closely linked to the land is consistent: “I just think being here is enough culture” (C6). Other clients spoke of feeling the “old people” such as: “You can feel them in some rooms. They’re probably just looking for someone or passing through” (C5). The ancestral connection to the country on which OH is located, including being at a known traditional meeting place and where respected Elders once lived, was also noted to be significant by staff. For example:

“I know a very well respected old Aboriginal man used to live here, used to own this place and they did a lot of cultural stuff. His house was over there [pointing]...” (S7).

“Somewhere along this river was a meeting place in days gone by, the people that lived here before Orana Haven were very, very cultural... being on the banks of a river has a cultural aspect for Aboriginal people, that’s where most of their meeting places are...” (S6).

Clients who had little connection to Aboriginal culture prior to coming to OH discovered that being on traditional country provided access to a range of cultural experiences:

“There’s a couple of fellas from the city and they’ve never been to a bush place. I suppose they’d like to learn what berries to pick and make you healthy and stuff like that. One fella didn’t know Aboriginal ways, but, he ended up getting the message… he started mixing in and doing things, following us around, [saying] “Oh yeah, I love this!”” (C3).

Examples of using the bush and the country to access culture included regular ‘culture trips,’ which was reported to involve looking for wood to make traditional artifacts such as boomerangs and nulla nullas (traditional Australian Aboriginal hunting and tools), visiting significant sites, and learning about traditional medicines. Importantly, the staff believed it was this access to culture from being on country that positively impacts clients’ health and wellbeing. Staff commented that it is their responsibility to impart knowledge about culture:

“We take them out bush, show them which trees have the fruit, which ones are the medicine. A lot of them like the medicine... There’s one medicine that’s good for your liver and a lot of them drink it when they’re here, they go and get it and we show them how to mix it. Some have pretty high liver counts and by the time they leave here and they’ve been drinking the medicine, the liver count drops” (S7).

#### Identity

Clients reflected on a range of positive experiences of learning and practicing their culture while at OH. One client described the process and persistence required to make, and learn to play, a didgeridoo (a traditional Australian Aboriginal musical instrument), which was reported to directly impact on his identity as an Aboriginal person:

“You sand it down. We’ve got the wood all cut up, just go and pick a good one out and clean it up. You give it a wipe over with paint, it gives it a shine or something, takes a lot of splinters away from it. Then you can carve it… Learning to play is something I do nearly every morning. I try but, I can’t do it, but one of the koori fellas said to me, “You can do it, just keep trying and you’ll get it. Just keep trying and it will work.” It’s like just sitting down playing the guitar... It keeps our Aboriginal culture alive, which I never knew how it was done as an Aboriginal person… I feel happy and proud about it” (C1).

This sense of pride and happiness that this client and others reported from making cultural artifacts or engaging in other cultural activities was perceived by staff to improve their sense of self-worth. For example an Aboriginal staff member stated:

“It’s given me a lot of joy seeing them making something like a didgeridoo. Watching their self-esteem lift up…“I can do this.” Because a lot of this stuff, I believe, the aiding of the self-esteem, self-worth… gets rid of the depression” (S3).

Staff consider a major indicator of the success of the program, therefore, was the strengthening of clients’ identity as Aboriginal men through actively engaging in cultural activities:

“A lot of fellas are lost or got no identity, don’t know where they came from. We understand where they’re not complete in their identity and that’s why we do these culture things, like the young fella that learnt to play his didge, he said, “Now, I feel like a blackfella”” (S3).

#### Spirituality

Clients and staff interchangeably used the term ‘spirituality’ to refer to both traditional cultural spirituality (e.g. “feeling the old people” (C9)) and more conventional forms of religious spirituality aligning with the 12-Step model, a foundation of OH’s program. Both clients and staff perceived OH had an appropriate focus on spirituality embedded in the program and it was viewed as a positive that spirituality could be individually personalised. For instance, one client reported they were encouraged to view his “higher power” to be any source of strength in his life, which he described being his sister and his son. A staff member explained how clients were encouraged to identify their own perception of spirituality;

“We tell them, “Your God or higher power can be anything, your partner, your children, it could be your great grandfather” (S8).

And that belief in a higher power was critical as this is consistent with traditional Aboriginal beliefs;

“A lot of us Aboriginal fellas, we say, “Baiame, is our God.” Everyone’s got to believe in something, if you don’t believe in something you’ve got nothing” (S8).

### Emotional safety and relationships

The importance of emotional safety, including trust, acceptance, feeling safe, and developing relationships was described by participants in relation to the following components: therapeutic activities; and staff empathy and lived experience.

#### Therapeutic activities

There were two types of therapeutic activities described by participants: groups (e.g. 12-Step, morning therapy and psycho-educational groups); and individual counselling (described as one-on-one time with a counsellor). A majority of clients perceived the therapeutic groups to be the most helpful therapeutic activity during treatment. Groups gave them an opportunity to share experiences about their problems, feel understood and learn from others experiences. For instance, one client said:

“The groups have been unreal... When staff are talking it’s like they’re taking everything out of my head and just saying it. It’s like they’re only talking to me, like I’m the only person in the room with the problem… It’s good to listen, just to sit there and listen” (C12).

This was found to be especially beneficial for clients who reported vulnerabilities with expressing themselves or their emotions. As such, a critical element of OH’s groups, as identified by clients, was that “you’re allowed to share if you want, but they don’t force you” (C10). Clients consistently reported a preference to listen when they first arrive, and then they gradually “talk more and more” (C3) each time as they build their confidence and trust within the group. Given this, trust was perceived as a very important enabler for clients to share their experiences, as they felt more accepted into the group:

“We’ll talk about our stress every morning instead of holding it in… We talk in a circle and we all know it stays in the room” (C1).

According to the clients, there were two types of individual counselling provided at OH. First, most clients reported receiving individual counselling from the visiting psychologist or psychiatrist at the local Aboriginal Medical Service (AMS). The focus or content of these sessions was not described. Second, several clients described “having a yarn” (C4) with OH staff in more informal settings. These clients reported feeling staff were approachable and were “always there to have a talk” (C1) both day and night when they were “worked up and need a chat” (C3) or felt like leaving the program.

#### Staff empathy and lived experience

All clients reported positive perceptions about the OH staff, stating “it’s not just a job for them… they go out of their way” (C7) and that staff are “always available for a chat… genuine, easy to get along with and feel caring” (C11). Staff reinforced the importance of providing client-centred care:

“We’re all on the same page, the whole purpose of the job is to help these fellas in their journey to do something different with their life” (S4).

Clients reported staff related to them better because they had “been there done that” (C11), meaning that they were aware that staff had lived experience of substance misuse which clients perceived as a strength of the program. One staff member reflected on how their shared experiences are similar:

“Since I’ve come here I’ve worked with a lot of the guys and there’s not much difference in our stories, the only difference is that some of the guys end up in jail, but all the traits were the same, all the habits, what we did was the same” (S3).

An example of how a staff member used their lived experience to relate better to clients was when talking to them prior to admission to OH, using their lived experience to reframe their understanding by feeling empathy for clients:

“I tell them that, “You’re going to do it hard… but if you use before coming, it will be harder.” But, I have to remember about my experiences, because, when I was going to rehab I would have used that much… to get that last hurrah” (S5).

Staff lived experience was also viewed by some clients as more important than formal qualifications:

“It’s pretty good here too because all the staff that are here have been through it, they’ve been in the same boat, whereas some of the city rehabs and that they’re just people out of university and that, so it wouldn’t work as good” (C10).

Staff with lived experience demonstrate by their example that recovery is possible, and sharing personal experiences in the group setting can reinforce this notion of recovery. For instance:

“Sometimes when I talk about the past it brings back a lot of memories, but when I walk out clients come shake your hand and say, “Thank you,” because they didn’t expect it” (S8).

However, a small number of staff reported university or other relevant training related to their role. One staff member reported that he progressed up the ranks through “on the job training” (S3):

“I started off as a residential care worker, I did cooking, cleaning, assessments... As each opportunity came, I grabbed it… I really enjoy it! I couldn’t imagine where I’m at, and since I’ve been here I’ve been offered training, I actually finished university last year, which I never, ever thought I’d be able to do, I never dreamt of it” (S3).

Staff additionally conveyed that identifying as Aboriginal or from the region helped to build rapport and develop cultural bonds with clients, and was perceived as a strength by both staff and clients:

“I think a lot of boys feel comfortable if you say, “Where are you from, brother?” I always ask them where they’re from, “Oh, do you know such and such,” because nine out of ten times they know the people we know…“Yeah, Unc, I know him... You know him?” “Yeah” (S4).

“I'm Ngemba... this country is Ngemba. My great, great, great grandfather, that’s how far I go back, he was the Chief of this tribe… I’m connected to this land strongly” (S8).

As a result of employing a mix of staff with lived experience, formal qualifications, links to the region or identifying as Aboriginal, clients and staff report mutual respect and genuine connection, which appears to reinforce a perception of safety that promotes healing. For instance:

“They feel safe around us, and when you know they feel safe around you and respect you, you start to see them healing” (S9).

Despite this, some staff reported challenges of working in a remote Aboriginal residential rehabilitation setting, including the difficulty of caring *too much* about the clients, for example:

“The managers say, “Look, be there, but you’re not their friend, you’re just a worker.” But it’s a hard when you’re with them for three months and you get to know them, and later someone says, “So and so got killed, so and so overdosed, this one’s got locked up”” (S4).

As a result, working in a “24/7 remote service with the most complex, disadvantaged members of society” (S6) was described as “mentally draining” (S3), with staff reporting feeling overwhelmed.

### Strengthening life skills

To ensure they can overcome substance misuse and lead meaningful lives when they return to families and communities, clients need to strengthen fundamental life skills. Rules and routine, program structure, and work-ready skills were reported to enhance the development of life skills.

#### Rules and routine

Overall, the rules and routine of the OH program appealed to both clients and staff. Rules are explained to clients when they first arrive in “plain language,” and read out each week to remind people of them. As described by clients, the rules are “strict, but fair” (C2), with one client stating that once they arrive, there is an acceptance that they are not coming to OH to “sit around for three months so it can look good for court” (C5). For example:

“They don’t let you just come here and have a free run, or a free ride, you’ve got to work for your sobriety, or earn it in a way” (C4).

The following staff quote articulates the benefits of discipline, inferring that the routine and rules embedded into the program are fundamental to the program and the client’s, success:

“I don’t know everything, but if you haven’t got structure you’ve got nothing, you’re just wasting your time if you haven’t got structure…rules and structure makes this place” (S4).

One particular rule that was considered strict but fair relates to routine urine screens. While clients reported being aware that a positive screen meant instant program discharge, staff also positively described clients wanting to see the results if they are clean, finding this helps maintain their progress:

“They say: “It’s good to see my results…this is the first time I’ve been clean this long.” It gives them confidence in themselves and lifts their self-esteem, their confidence…like they’re proud of themselves, some blokes walk out real happy. Some of them say, “This is what we need to do more of, it keeps me going”” (S3).

Another rule clients discussed was the expectation to have undergone withdrawal prior to admission. Over half of the interviewed clients reported formal withdrawal, with one client presenting to the hospital “drunk with 15 cans” (C2) Clients described withdrawal as “hard and that it felt like I was dying” (C4). Clients reported being pleased to have withdrawn from substances prior to coming to OH.

Despite having firm rules in the program, staff also reported some flexibility to ensure each client’s needs are considered before decisions are made. For instance:

“You can’t approach everyone the same, it’s not a cannery, it’s not jail. We are individuals and we care, so you can’t give everyone exactly the same approach… If you’ve got too many people then it just becomes a cannery” (S6).

Staff consistently reported that the routine and the program rules have been designed with a purpose that aims to promote long-term healing from substance misuse after the clients leave OH. An example of this includes the gradual increase in freedom as clients achieve set time in the program:

“It’s really clever how the program set up, there’s a purpose for everything…. I just love the whole three months! The first month they’re not allowed to go to town because they’re really finding it hard to just deal with stuff emotionally, the second month they’re allowed to go shopping… gives them that little bit of freedom. They might see dealers down the street and they have to say, “No”” (S9).

Structured activities on the weekend aimed to encourage clients to develop hobbies, as weekends are typically high-risk times for substance misuse:

“On weekends clients don’t often have a real lot planned and that’s to try to help them start to learn to do their own thing… because when they go home, the weekend is their hardest time, so it teaches them to learn to occupy themselves” (S9).

Following a consistent daily routine and developing personal responsibility was perceived as a vital life skill to continue when clients returned home or gained employment. For example;

“Some don’t get out of bed until two or three… We say, “You go to bed at a certain time, have plenty of rest, get up at a certain time, attend group on time. I tell them, “What if you go to the doctor and you’re late, what happens?” We’re trying to get them into routine, be punctual, be responsible. It gives them structure. When you’re in an addiction, the whole household is in chaos, so when they go home they can put a bit of structure back” (S3).

Finally, the value of routine and structure was connected to Aboriginal culture to ensure a deeper understanding about why this is an important skill for clients to strengthen:

“Things come up about the Aboriginal people and I say, “Why do you think they survived? They had structure, routine, that’s why they survived, and they followed it… that’s what you’ll learn here. Work out a structure and follow it.” Anything I can find that links back to Aboriginal culture… hopefully it triggers something in them” (S3).

#### Opportunities to develop work-ready skills

All clients described partaking in two or more work-related activities while at OH, including rebuilding small engines, woodwork, developing literacy, numeracy and financial skills, or volunteering (e.g. building garden beds for the local school). Participants reported very positive perceptions of their experiences of the work-related activities, with most stating that it “keeps you busy” (C1), making “the time go fast” (C5) which resulted in “less time to worry” (C2). Most importantly, clients reported they enjoyed “learning new things” (C1) with another stating he “wouldn't be learning all of these things if [he] was back home” (C10). Completing vocational courses enhanced their sense of pride and confidence, exemplified by one client describing how he felt after learning to rebuild an engine:

“I’m very proud of myself, I thought I couldn’t do it, I had the biggest smile” (C1).

Several staff also conveyed that learning practical skills helps clients in their everyday life not just for attaining employment, such as completing a small motors course to fix their own vehicles rather than paying for a mechanic. Learning work-ready skills was perceived to achieve three outcomes for clients. First, it empowered clients and built self-confidence; second, it reduced their time being “idle-minded” (C8); and, third, it increased their sense of “personal responsibility” (C6) to seek employment after finishing the program.

### Improved wellbeing

Participants discussed observable physical and mental changes as clients progressed through the program. Abstinence and observing improvements in their overall wellbeing allowed clients to cultivate hope for a better future for themselves and their families.

#### Client trauma and improvements to mental wellbeing

All clients reported experiencing trauma, ranging from one-off events to prolonged exposure. The following quote comes from an Aboriginal man who had been drinking for over 30 years:

“I lost my mum and hit rock bottom… I think round about the same time they came and took my three young boys and to numb the pain I turned to alcohol. I would drink every day (C2).”

Descriptions of mental health problems were common. The following examples provide a powerful insight into the relationship between mental state and substance misuse:

“Mental health forced me to drink because I’d forget about everything, and then it would all just flood back to me when I had too much grog and I’d try to kill myself all the time” (C3).

“Without my medication I wind right up and get faster and faster and faster in my thought patterns and it won’t slow down, but on my meds it slows me down. That’s why I drink when I’m off my medication, to slow me down, because I hate my brain; it terrorises me” (C6).

Most participants reported limited access to or knowledge of services that provided mental health treatment. Since being at OH, and having access to health services, several clients reported feeling like they were now “thinking clearer” (C4) or that their life felt like it was “back on track” (C7). For example;

“I used to be always thinking…24/7. Now I’ve slowed down to 10 minutes daily” (C4).

“It’s a breath of fresh air - good thinking time. You can really get your life sorted here” (C6).

The location of OH was described as a major factor in improved wellbeing, with clients commenting on a love for being in the bush and that OH is “sort of like coming home” (C2) or that being at OH is “safe and peaceful” (C1). For instance;

“I just feel comfortable out here, there’s something about the place, once you get settled in, there’s something about it” (C7).

Several clients specifically stated being near the river to be important to them. For example;

“I love the river. It’s a big relief for me to be on the river – all of a sudden just go for a walk down the river or something and just clear your head, it helps a lot of us boys” (C4).

A number of clients referred to the remoteness of OH and being near a local community as important because they “had heard a lot about” (C2) the country or they felt more comfortable due to being in closer proximity to their family rather than being in a place that felt unfamiliar to them.

A good “balance of downtime and activities” (C9) was considered as a strength of the program, with one client reporting: “There’s a lot more spare time here compared to other rehabs, this place is not in your face” (C4). These comments were also consistent with staff perceptions about the location and activities combining to give “clients a break from the full on” (S2) and valuable time away from substances and chaotic lives at home to process their experiences;

“The benefit of this place to these fellas is time out, it gives them time for their minds to become a little bit clearer and start thinking about the rest of their life. Until their mind is clear [from substances], they can’t think” (S7).

#### Improvements in physical wellbeing

Improvements in physical wellbeing were regularly reported as being necessary to a client’s overall progress towards healing. One staff member recounted their observations when clients first arrive:

“When they come, some have no colour in their face, very little eye contact, no confidence, not steady on their feet… very thin” (S3).

Most clients described improvements in their physical health since being admitted to OH, with one client reporting that his family immediately noticed the difference in his appearance: “I feel good on the inside and I feel good on the outside” (C2). Putting on or losing weight was a major indicator for good health for the clients, especially those who were not eating while using substances, for instance: “Just when I first stepped here I only weighed 55 kilos and I weigh nearly 90 kilos now” (C8). Some clients also reported sleeping better than before coming to residential rehabilitation. For example: “It feels good to wake up every morning without getting drugs” (C8).

Food and nutrition were also perceived as a fundamental component to both physical and mental wellbeing, with several clients reporting how food has helped them in their healing process from substance misuse. Clients reported “eating better than in jail” (C12); that their appetite increased or that they enjoyed eating regular, healthy meals. Similarly, OH staff reported that they can see client’s “getting their taste buds back” (S7) after a few days in the program and often observe improvements in their appetite, their “glowing skin” (S3) and gaining weight.

#### Hope for a better future

Abstaining from substance misuse improved overall health and wellbeing, facilitated clients to set goals and provided a glimpse of a better future.

“The longer you’re off it, in a way, the more chance you’ve got. It’s a good program, it seems to be working [for me]” (C10).

“I’ve learned heaps about why I’ve taken drugs and all that sort of stuff, it gives me a strength, not to go back to the outside and start the whole cycle again… I just think about goals to set for when I get out” (C2).

This perception of hope is derived from a range of goals discussed by clients, such as wanting to be a role model, wanting to be a better father, and hope for their relationships. For example:

“Once I got locked up this time, I said, “This is it, it’s time to throw everything away.” It was the second time and I was thinking to myself, “I don’t want to keep doing this, it’s not the life I want to give my children”” (C1).

A staff member also reflected the hope observed in clients:

“I see them wake up… have hope in their eyes. Hope is the biggest thing I see for the fellas, and we’re talking about fellas who have been in and out of the correctional system for years, and they think that’s normal. They’ve seen their parents go through domestic violence and they’re passing it down to their kids without realising what’s going on. When they come to Orana, they start to think, that maybe they can intervene” (S6).

### Perceived areas for improvement

Clients and staff perceived a range of areas for improvement in the current provision of OH treatment, namely for a greater focus on aftercare, more access to resources such as mental health services and improved understanding of the value of culture in treatment.

#### Aftercare

Staff reported typically providing details for local health services and Alcoholics Anonymous meetings in NSW to clients prior to discharging, in addition to limited personalised support such as monthly phone calls. Both staff and clients, however, strongly identified that more structured or robust aftercare support could be useful in reducing rates of relapse to substance use and readmission to OH. However, despite this, clients typically described feeling uncertain about what supports would be available to them when they left OH and returned back to their community: “Not sure what I’ll do, take it day by day, I guess” (C2). Staff also identified major limitations with OH’s aftercare support, as they were aware of the increased likelihood of relapse and re-admission if partners or family members continue to misuse substances and/or do not support the client’s decisions to maintain their progress, for instance:

“If you haven’t got support it’s very hard, especially if your girlfriend or wife are using; what chance have you got? (S7).”

Another staff member provided a powerful insight into the client struggle once they are discharged:

“We had a bloke from Moree and after three months he was clean, looked well, like a billion dollars. When I took him home, I dropped him at a beautiful home, beautiful wife. He rang me up a couple of weeks later drunk, crying, wanting me to take him back, and I said, “What happened mate?” and he said, “I did what you said not to do. I started hanging around when my mates were drinking,” and now he hasn’t got a house because his wife won’t have him while he’s drinking. He’s living homeless until he can get back in here” (S4).

Staff therefore suggested two solutions: referrals to designated workers in each community, and incorporating the client’s family in their transition home.

#### Access to additional resources

Clients identified barriers to accessing mental health treatment. For example:

“There’s sometimes anywhere from 10 to 15 people out here, so there’s 15 other people that have been in jail that need to see psychologists and psychiatrists, especially some of the boys that have been in jail more than two years because it would be pretty tough. So, you just pretty much have to wait until there’s a vacancy really” (C8).

Staff also believed that the current level of resources provided to OH was a barrier to providing an effective program for clients with mental health problems;

“We can only do so much for their heads at Orana, but some of these guys need more mental help while they here and after they leave” (S3).

Staff also highlighted pressures between balancing case management tasks (e.g. file notes, referrals, referrals, weekly scheduling) and time for one-on-one work with clients, in addition to managing expectations from the community. For instance:

“There’s a lot of things you’ve got to coordinate, expectations from community, expectations from funding, expectations of staff, expectations of your residents; there is a lot of expectation around these places” (S6).

Other concerns related to lack of resources included: ongoing costs associated with isolation such as cost of food and transport to OH; and the costs in time and money to attend meetings or training. While the location was perceived as a positive overall, the costs associated with remoteness were not reflected in the budget for the program.

#### Better understanding of the value of culture in treatment

Clients reported a preference for more cultural activities (e.g. smoking ceremonies, camping, bush medicine), with one client suggesting a full-time cultural Elder would be beneficial because it was “important to remember what happened in the past” (C12). Staff also identified a need for more cultural activities (e.g. “sometimes I don’t think we do enough culture here, we need to make sure that all the stuff we do leads back to culture” (S5)); in addition to a perceived disconnect between policymakers, funders and NSW Aboriginal residential rehabilitation services about the value culture has in the residential treatment setting. For instance;

“Bureaucrats don’t understand the importance and time it takes to work with the boys about their culture” (S6).

Therefore, staff emphasised the need to have a better understanding of the value culture plays in healing during residential rehabilitation treatment.

## Discussion

This paper identified that Aboriginal drug and alcohol residential rehabilitation is not just about length of time in treatment, but also about the culture, activities and relationships that are part of the treatment process that enables the client to change over time. This study found that cultural elements of the program were highly valued by both clients and staff of a remote Aboriginal residential rehabilitation service, with the country or location being fundamental to the daily practice of, and access to, culture. Secondly, specific factors such as trusting and connecting with staff, consistent rules and routine, gaining work and recreation skills, and improved wellbeing, were considered program strengths. Thirdly, this paper identified areas for improvement including aftercare support, more access to resources such as mental health services, and the need for a better understanding of the value of culture as treatment.

### The value of culture

The cultural component of Aboriginal residential rehabilitation programs is the point of difference between these programs and non-Aboriginal rehabilitation services. Connection to culture is perceived as critical to recovery from substance misuse by staff and they facilitate connection through location, cultural activities and education. This finding is consistent with papers outlining that recognition of culture in Aboriginal drug and alcohol programs are critical [14, 48]. The ‘culture as treatment’ hypothesis coined by Brady [15], refers to this return to traditional cultural practice for improved wellbeing, and while it has since been supported conceptually in the literature [23, 55], its measurable impact continues to remain an open empirical question [31]. Nevertheless, results from a recent meta-analysis of cultural adaptions of psychological treatment programs are promising: they identified that culturally adapted treatment for Indigenous people had almost five times greater likelihood than other treatment to engender remission from psychopathology [58]. The current study demonstrates that embedding culture within the program ensures clients have opportunities to acquire a meaningful connection with their heritage and strengthen their identity. The delivery of the program by Aboriginal staff with similar experiences of substance misuse is critical in cultural connectedness, trust and therapeutic alliance [1, 20]. The integration of culture into routine service delivery appears preferable to implementing a single program component of ‘cultural activity’ because culture is not an activity at OH but a philosophy of change [14, 22, 61].

### Therapeutic alliance

Staff empathy and lived experience established real life examples for clients of how recovery is possible. In previous studies, positive therapeutic alliance is associated with higher rates of retention in treatment and improvements in post-treatment outcomes [17, 41, 50, 60]. While therapeutic alliance is not measured in this study, participants provided many examples of trusting staff, believing in their authenticity and perceiving staff knew what they were doing. Further, ward atmosphere, a phenomena shaped by the social structures and interactions in the caring environment, has been previously found to enhance retention in both psychiatric and substance abuse programs. Moos [43] describes three specific dimensions of ward atmosphere: relationships (involving aspects of support and quality of personal relationships); personal growth (the level of encouragement for personal change and development among patients); and system maintenance (how well ordered and organised a ward is). These dimensions were found to be reflected in participant statements.

### Developing life skills

A third perceived strength was the value of developing skills for after discharge. The rules and routine, planned activities (e.g. groups, work-ready education, cultural activities, chores) interspersed with rest and recreation, were perceived as important. Evidence from previous research supports this finding, with a large-scale study of residential treatment services in the United Kingdom identifying higher completion rates associated with the provision of a balanced treatment program that is not too demanding [41]. Additional research recommends developing conventional social routines (e.g. routine sleep times) increased the likelihood of treatment compliance [59]. Nevertheless, in Aboriginal drug and alcohol residential rehabilitation settings, a paradox may still exist for a service to be both flexible and structured, as some clients seek residential rehabilitation for a short-term break from substance misuse primarily for recuperation, while others are seeking more structured treatment-focused activities, such as strict rules and routine [14, 61].

### Perceived improvements in health

A return to better health was also established as a key strength of the program and an integral aspect of the healing process for clients. Perceived improvements of health after a period of abstinence is correlated with positive self-evaluations of overall quality of life, mental health, reduced stress, and hope [21, 29, 52, 67]. Similarly, inmates in a correctional facility who had perceived their health to be poor prior to incarceration were more likely to report improvement in their physical and mental health during incarceration [65]. However, without continuity of care upon release, any health improvements, real or perceived, are likely to dissipate when released [65].

### Perceived areas for improvement

Both clients and staff perceived aftercare as a major area for improvement for OH. Research indicates that the post-treatment period is when a client is most vulnerable, and requires more attention to regularly monitor their status, for early detection of potential problems, referral to appropriate services, and re-admission as required [38, 51, 65]. Evidence demonstrates that a lack of aftercare is not unique to Aboriginal residential rehabilitation services, but that people with chronic substance misuse typically receive single episodes of treatment [27, 38, 39]. This is especially important given the risk of relapse. Clients may have reduced tolerance as a result of abstinence from substance use, which can increase the risk of death or serious harm if a client relapses [65]. Aftercare support has been found to improve treatment outcomes the longer the duration of aftercare [42] and the more family is involved [18, 48]. The length and types of supports provided for aftercare should also be dependent on the complexity of issues facing the client upon discharge [38]. It is therefore recommended to strengthen the continuum of care for OH clients by developing an evidence-based aftercare support program as part of an integrated model of care.

A second perceived area of improvement focuses largely on resources in residential rehabilitation treatment to provide better access to mental health services and support staff to access adequate training. A lack of resources to maintain effective, evidence-based services have been consistently reported for decades [14, 20, 32]. Lastly, staff identified the importance of empirically evaluating the impact of ‘culture as treatment’ in future research with Aboriginal residential rehabilitation services. Such evaluations would contribute to improving the understanding of the impact of culture in residential rehabilitation treatment, which was identified by staff to be lacking amongst many policy makers and funders. Further, creating partnerships to evaluate programs between services and researchers represents best-evidence practice [25].

### Limitations

This is the first qualitative study to empirically analyse the stories of both clients and staff in a remote Australian Aboriginal drug and alcohol residential rehabilitation setting. While this study makes a unique, qualitative contribution to drug and alcohol literature, there are some limitations. First, the interviews were conducted in a single setting, and the findings relate specifically to OH. Second, the interviews were conducted with clients attending the OH program, which therefore increased the potential risk for bias, as researchers did not include the views of clients who had discharged from the program. Third, the interviewer was non-Aboriginal, which may have impacted on the richness of interview data. Finally, the IPA methodology used to analyse the data focused on lived experiences of OH to identify strengths and weaknesses of the core program components, which restricted deeper analysis about why staff and clients valued specific components over others.

## Conclusion

Through listening to the stories of clients and staff, this research has identified that Aboriginal drug and alcohol residential rehabilitation is not just about length of time in treatment, but also about the culture, activities and relationships that are part of the treatment process. This study found that cultural elements of the program were highly valued by both clients and staff of a remote Aboriginal residential rehabilitation service, with the country or location being fundamental to the daily practice of, and access to, culture. Developing reliable and valid assessments of the program components of culture and treatment alliance would be highly useful, given this study has reinforced their perceived importance in achieving positive treatment outcomes. Further, clients and staff identified that strengthening the aftercare program, as part of an integrated model of care, would likely provide greater support to clients after discharge.
